# Editorial: Insights in neurocognitive aging and behavior: 2022

**DOI:** 10.3389/fnagi.2024.1361839

**Published:** 2024-01-16

**Authors:** Kristy A. Nielson, Annalena Venneri, Shin Murakami

**Affiliations:** ^1^Director, Aging, Imaging, and Memory (AIM) Laboratory, Department of Psychology, Marquette University, Milwaukee, WI, United States; ^2^Department of Life Sciences, College of Health, Medicine, and Life Sciences, Brunel University London, London, United Kingdom; ^3^Department of Medicine and Surgery, University of Parma, Parma, Italy; ^4^Department of Foundational Biomedical Sciences, College of Osteopathic Medicine, Touro University California, Vallejo, CA, United States

**Keywords:** cognitive aging, brain aging, Alzheimer's disease, neuroimaging, biomarkers, cognitive reserve, lifestyle, exercise

Major advancements in the fast-growing field of neurocognitive aging and behavior were highlighted in the inaugural *Insights in neurocognitive aging and behavior: 2021* Research Topic. It included 15 articles that addressed novel approaches to identifying and predicting cognitive decline, neurocognitive markers for Alzheimer's disease (AD), lifestyle contributions to aging and AD, and commentary on neurocognitive aging theory.

In this second edition, *Insights in neurocognitive aging and behavior: 2022*, we sought to build on the prior issue by highlighting the latest advancements and challenges for research in neurocognitive aging. Here we outline the contributions and implications for future research of 32 articles that addressed three primary topics at the forefront of neurocognitive aging research: (1) neural correlates and markers of cognitive decline; (2) age-related vulnerabilities, predictors, and differential diagnosis; and (3) modifiable factors and interventions.

## Insights in neural correlates and markers of cognitive decline

### Neural connectivity indices in neurocognitive aging

Over the past three decades, neuroimaging has transformed how we study and understand neurocognitive processes. The study of cognitive aging and dementia has been dramatically influenced by these developments. Important advances in neuroimaging have emphasized measures of connectivity amongst brain areas during rest and while performing various tasks, and as a function of individual differences. These measures employ advanced technologies, including (among others) magnetic resonance imaging (MRI), functional MRI (fMRI), and electroencephalography (EEG). Two articles pursued methodological connectivity advances relative to aging. Chuang et al. used fMRI to directly compare functional connectivity (FC) and effective connectivity (EC) indices as predictors of cognitive and brain health in middle age. FC is the synchrony (i.e., correlation) between regions, while EC attempts to account for causal, indirect influences of source regions on target regions. Their results for resting state showed EC was greater in younger age, alcohol abstinence, lower body-mass index, fewer white-matter hyperintensities, and better delayed memory and reading. By comparison, FC was greater in women and abstainers. Task-related EC was greater in those with lower blood pressure, fewer white matter hyperintensities, and better vocabulary (especially in women and White people). Task-based FC was greater in women, with low genetic AD risk, and in those with better working memory. Thus, resting and task FC and EC provided different, complementary indicators of brain and cognitive health, with particularly valuable contributions of FC to understanding the flow of information processing. To index global cognitive health, Tobe et al. used a different approach; they used resting EEG and graph theory analysis to identify an activation hub and related whole-brain interactions. Better cognitive functioning was apparent with greater frontal lobe whole-brain interaction specifically in the theta band, which is associated with top-down cognitive control. They suggest this approach could effectively index global cognitive health in longitudinal and intervention studies. Indeed, the results are consistent with other neurocognitive findings linking successful aging and neurodegenerative disease to the efficacy of frontal lobe network function and connectivity (Paitel and Nielson, 2021, 2023; Di Tella et al., 2023).

Five additional articles in this Research Topic examined applied aspects of neural connectivity in aging. Xia et al. reviewed current evidence of structural and functional brain connectivity related to age-related decline of cognitive control that leads to loss of independence and quality of life (Clark et al., 2012; Yagi et al., 2020). They concluded that maintaining brain structural integrity is essential for functional neural compensation against age-related changes in these networks, suggesting that future multi-modal studies should focus on maintenance of neurostructural integrity as an outcome of independent living and quality of life. Complementary recent research employed transcranial alternating current stimulation to directly alter functional connectivity, which effectively improved cognitive control (Jones et al., 2022). Tsai et al. examined hippocampal connectivity in both subjective cognitive decline (SCD) and migraine, which are often co-morbid. They found both SCD and migraine patients had comparable altered connectivity between anterior and posterior hippocampal regions and cortical areas relative to controls, suggesting overlapping etiology. Using multivariate analyses of resting fMRI, Goelman et al. examined sensorimotor functional connectivity. Young adults had unidirectional functional pathways from primary motor/sensory cortex to higher cognitive areas, while older adults had more complex pathways that suggested slower, less efficient processing in elders. Indeed, older adults with pathways resembling young adults had more education, consistent with better neural preservation (i.e., neural reserve, Nyberg and Pudas, 2019) and cognitive preservation (i.e., cognitive reserve, Stern et al., 2020). Montemurro et al. examined the influence of education on resting functional brain connectivity and cognition. Older adults with greater education had language and executive functions that were comparable to young adults, as well as comparable connectivity in multiple brain networks. The results suggested education affords better neural compensation (Nyberg and Pudas, 2019), resulting in better functional compensation in older age (Reuter-Lorenz and Park, 2014). Finally, Gutierrez-Zuniga et al. evaluated older-adult frailty as a function of brain volumes and white-matter connectivity. Independent of age and sex, frailty was associated with smaller cortical and subcortical volumes and poorer white matter integrity. However, these effects were due to specific frailty metrics, including polypharmacy, general health perceptions, and functional difficulties. Together these studies highlight the importance of progress in neural connectivity metrics for understanding age-related cognitive decline.

### The cerebellum in neurocognitive aging

Morphological changes in the cerebrum are the typical focus of study in aging research. Despite a growing appreciation for the importance of the cerebellum in cognition generally (Stoodley and Schmahmann, 2010) and cognitive aging specifically (Paitel and Nielson, 2023), morphological changes in the cerebellum in aging are seldom studied. Stalter et al. sought to address this gap, examining cerebellar gray matter volume in young and middle-aged adults, prior to the gross atrophy that is typical of older age. Middle-aged adults had smaller right posterior lobe (crus I/II, lobule VI) volumes, which are important in cognitive functions as part of the frontoparietal network, which may support the idea that cerebellar dysfunction may precede cortical changes in aging (Filip et al., 2019). Additional studies comparing cortical and cerebellar changes will help to confirm this hypothesis. Mo et al. examined the interplay between cognition, gait and balance disorders, functional brain connectivity, and regional cerebellar atrophy in older adults with cerebral small vessel disease (CSVD). Those with gait and balance disorders had poorer memory, greater cerebellar atrophy, greater cortical activation, and decreased cortical connectivity with the right posterior cerebellar lobe (VIIIa). Together these studies might suggest that the cognitive regions of the cerebellum are key early targets for aging-related interventions.

### Neural mechanisms and markers of cognitive decline in aging

Although studies of age-related changes and differences in brain morphology have been a primary focus of aging research, they often lack detailed cognitive assessments and examine only old age. Studies in this Research Topic attempted to address these issues and to examine brain morphology and brain activity in potentially important new ways. Clifford et al. compared brain volume changes in young, middle-aged, and old mice to promote translational findings to human models of aging. They found smaller isocortical volumes and larger subcortical volumes and white matter tracts in older age and with poorer working memory, similar to what is shown in humans. Yet, the greatest differences were between young and middle-aged mice, with smaller but significant additional differences between middle- and old-age animals. This trajectory diverged somewhat from human studies, suggesting that normal mouse aging trajectories differ somewhat from human aging. In a large sample of community-based middle- and older-age adult humans, Li W-X. et al. found white matter integrity was significantly related to visuomotor processing speed, semantic memory, and executive functioning after controlling for age. Poorer global cognition was also associated with smaller cortical thickness in several regions, independent of white matter integrity. Li M. et al. investigated blood oxygen level-dependent (BOLD) activity in white matter as a new, complementary approach to understanding functional brain organization. They found reduced white matter BOLD and reduced functional white matter connectivity with older age, indicating an age-related reduction in information exchange across remote brain regions. They suggested white matter BOLD as a sensitive imaging marker of neurocognitive aging. In another new direction, Sheng et al. overviewed neuroimaging analyses of gut-brain communication pathways toward highlighting the need for intentional multi-modal neuroimaging-omics studies. They emphasized the need to better determine the specific contributions of gut microbiota to the pathogenesis of AD and potential intervention targets against AD. Taken together, these studies suggest that neural morphology, connectivity, and brain-body communication changes may onset by middle age (cf. Murakami et al., 2011), that these early changes can index the integrity of cognitive functioning, and that they have potential predictive value for future cognitive functioning.

Two studies examined the role of pro-inflammatory and oxidative stress factors in cognitive functioning in aging, factors that are receiving increasing attention in the etiology of cognitive decline (Murakami et al., 2011). Ferreira et al. examined the iron-trafficking protein lipocalin-2 (LCN2), which is known to play a role in regulating neurophysiological functions related to neurogenesis, and has been proposed as a marker of neurodegenerative disease progression. They evaluated how LCN2 contributes to normative cognitive aging in mice. Mice who lacked LCN2 had reduced anxiety, but sustained depression-like behavior from a young age, accompanied by reduced hippocampal plasticity, which contributed to age-related cognitive deterioration. Since oxidative stress is amplified by the lack of LCN2, these results add to prior research to suggest the importance of reducing oxidative stress to promote healthy cognitive aging. Mrowetz et al. assessed key components of the synthesis of leukotrienes, a pro-neuroinflammatory factor regulated by microglia, as a potential marker of cognitive decline or resilience in aging. They found more microglia in older rats, with greater 5-Lox expression and 5-lipoxygenase-activating protein, key factors in leukotrienes production, in the more cognitively impaired aged rats. The results suggest that leukotrienes in aged brains might significantly contribute to cognitive decline, and that agents that reduce leukotrienes activity might promote better cognitive function and resilience in aging.

## Insights in age-related neurocognitive vulnerabilities and differential diagnosis

### Age-related neurocognitive vulnerabilities and predictors

The number of vulnerability factors, such as genes and diseases, that provide pathways to cognitive decline and AD is increasing (Murakami and Lacayo, 2022). Several studies in this Research Topic examined specific cognitive and brain network vulnerabilities in typical aging and dementia. Li N. et al. studied visuomotor adaptation, a key ability for independent living, to better understand why it declines with age. Comparing younger and older adults on both manual and eye-tracking performances, they found poorer visuomotor adaptation in older age occurs due to combined, but separable changes in motor anticipation and motor execution. Cai et al. tried to clarify the link between muscle strength and cognition in older adults. Greater handgrip strength (with age and sex covaried) was positively correlated with working memory and prefrontal cortex activity, although neural activation did not mediate the task by grip strength relationship. Ren et al. examined punishment frequency to determine how age-related changes in risk and reward processing influence decision-making. On the Iowa Gambling Task, older adults were effective in making advantageous decisions, but they often failed to avoid disadvantageous decisions. The results suggested that older adults may have elevated sensitivity to risk when punishment frequency is high, and thus they make poorer decisions, due especially to overactivation in middle and superior frontal cortices, primary sensory, and attentional alerting networks.

With respect to vulnerabilities to dementia, African Americans (AA) have an elevated risk of AD in general (Rajan et al., 2018) and even greater risk when carrying the Apolipoprotein-E ε4 allele (Farrer et al., 1997). Obesity is associated with greater AD risk in midlife (Whitmer et al., 2008), but less is clear about it in older age. Thus, Osiecka et al. examined the role of ε4 and obesity in cognitive functioning and structural-MRI measures in older AA (86% female; 39% ε4+). After controlling multiple covariates, most cognitive variables did not differ by weight category or ε4. However, obesity in non-ε4-carriers was associated with larger hippocampal volumes, while obesity in ε4-carriers was associated with smaller hippocampal volumes. Thus, in older AA, obesity may be protective of hippocampal function, except in those with genetic risk for AD, where obesity amplifies risk.

### Contributions to differential diagnosis

The clock-drawing test is often used in dementia assessment batteries. Kehl-Floberg et al. assessed whether free-drawn clock-drawing tests (Rouleau system, clock drawing interpretation scale) could effectively detect subtle cognitive decline in a large sample of community-dwelling older adults. They found both scales were clinically relevant and informative in larger test batteries, but neither was suitable as a stand-alone assessment or screening tool for subtle impairment. Wang et al. analyzed subscores of the Mini-Mental State Exam (MMSE) attempting to distinguish hypertension (HYP), cerebrovascular disease (CVD), and coronary heart disease (CHD) patients using network analysis. HYP had reduced network integrity for time orientation, delayed recall, repetition, and reading. CVD had poorer network integrity for memory, spatial orientation, and general cognition. CHD had sparse cognitive networks across multiple cognitive functions. The results suggest differential cognitive vulnerabilities across cardiovascular disorders that could be valuable for diagnosis. Relatedly, Li X. et al. found the severity of cerebral microbleeds in CSVD was predicted by attention and executive functioning, suggesting these metrics are an important part of diagnosis and tracking over time. Keszycki et al. focused on neuropsychiatric phenotypes as possible predictors of tau neuropathology in neurodegenerative disease. They found the combination of apathy and irritability significantly predicted tau pathology in frontal-temporal lobe dementia (FTD), while sleep but not appetite disturbances were predictive of progressive supranuclear palsy (but not FTD). They concluded that neuropsychiatric profiles can contribute to differential diagnosis and prediction of underlying neuropathology. Finally, Branch et al. evaluated odor memory in rats to determine whether it could serve as a within-subjects, repeatable index of hippocampal integrity. Compared with young rats, long-term recall of odor cues in old rats was related to hippocampal function (i.e., spatial memory), and this was stable over time. Thus, novel odor recognition may index hippocampal integrity and be valuable for evaluating cognitive improvements in human intervention studies.

## Insights in modifiable factors in neurocognitive aging

### Lifestyle and environmental factors as interventions

Lifestyle behaviors and activities (e.g., diet, exercise, social engagement) are a type of modifiable factor that can protect the brain and cognition from age and neurodegenerative disease. Indeed, they might reduce the risk of dementia by 40% (Livingston et al., 2020). There is rapidly growing evidence that physical activity or exercise can enhance neurocognitive functioning, widespread network connectivity, and white-matter integrity in both cognitively healthy older adults and those with cognitive decline (Konwar et al., 2023; Won et al., 2023). Santiago and Patashkin's review specifically suggests that physical activity may be an effective treatment or preventative strategy for neurodegenerative diseases. Specifically, they proposed the use of multidomain interventions that include physical activity, a healthy diet (e.g., Mediterranean), cognitive training, and good sleep hygiene. Lee et al. empirically evaluated a 10-month multidomain intervention consisting of group sessions of aerobic exercise (90 min once per week) performed concurrently with a cognitive task (i.e., “COGNICISE”) and twice monthly small group mentally stimulating social activities. In community-dwelling elders with mild to moderate cognitive decline, the intervention produced cognitive and physical improvements relative to controls who engaged in health education sessions. Together these studies suggest the need for more extensive use of multidomain interventions for older adults with cognitive decline.

Sounds that are task-irrelevant but environmentally meaningful have been shown to increase alertness and enhance perception, memory, and motor function performance in older individuals (Schwalbe et al., 2023). Manelis et al. evaluated this sound facilitation effect as a possible intervention by introducing sound during a modified Simon task, which examines cognitive control during interference. fMRI showed the sound facilitation effect was due to sound-induced sensory and attention-related cortical activation, which was attributed to neural resource recruitment to increase attention and alertness during the task. Thus, they suggest sound facilitation is a promising intervention. Pommy et al. reviewed fMRI studies of mindfulness meditation to clarify the neurovascular mechanism(s) underlying its cognitive benefits in older adults. They identified three potential mechanisms as targets for future research: (1) increased resting-state cerebral blood flow (direct neurovascular mechanism); (2) increased functional connectivity within the default mode network (indirect anti-neuroinflammatory mechanism); and (3) a top-down control mechanism influencing both direct and indirect neurovascular pathways.

Environmental enrichment is a non-pharmacologic intervention known to reduce depression and improve cognition in animals (Gubert and Hannan, 2019). Since maternal sleep deprivation results in depression and impaired cognition in adult rodent offspring, Zhang et al. evaluated whether these effects persist into older age in mice. They indeed found persistence into old age, but also that long-term environmental enrichment reversed these symptoms due to increased hippocampal neuroplasticity and reduced neuroinflammation. Ji et al. examined smartphone use in 5,000 older adults over age 60, finding that it was associated with reduced depression, which was strongest in males, participants over age 70, and urban dwellers. Further, the effect was mediated by engagement in social activity (e.g., political activism, volunteerism, leisure activities), suggesting the use of smartphones in elders to increase social and lifestyle activities. Together, these studies add to the rapidly growing literature showing that social and environmental engagement are important targets for reducing depression in older age (Manca et al., 2022).

### Cognitive reserve

CR refers to individual differences in resilience in the face of brain aging and neurodegenerative disease (Stern et al., 2020). Indeed, recent studies have linked CR specifically to efficacy of basal ganglia and frontal-parietal executive network connectivity and especially frontal network function (see also Di Tella et al., 2023; Montemurro et al.). CR is often indexed by educational or verbal proxies. Yet, this could underestimate CR in subjects with low education. Corujo-Balanos et al. addressed this concern using the Block Design subtest of the Wechsler Intelligence Scale as a possible non-verbal CR proxy. Although Block Design correlates with education and other crystallized intelligence measures, it non-verbally assesses reasoning and problem solving that is not a function of knowledge or education. They found a significant positive correlation between verbal CR measures and Block Design, suggesting that it is a suitable non-verbal proxy of CR. Peitz et al. evaluated whether being bilingual has long-term benefits on brain function relative to monolinguals. Longitudinal trajectories of gray matter volume and surface area suggested that bilingualism contributes to enhanced brain reserve (i.e., resilience), thereby leading to better CR.

## Conclusions

The articles in this Research Topic offer a glimpse at the new insights and possibilities for early detection of risk for cognitive decline and a better understanding of the etiologies of decline. They also provide insights into new directions in preventing or ameliorating age-related cognitive decline, including approaches that might reach high-risk and underserved populations. These studies also show the increasing and inevitable merge of neurocognitive research across neuroimaging, clinical, physiological, behavioral, cognitive, and social disciplines toward a better understanding of the whole organism, and better prediction and intervention in typical and atypical cognitive aging. As we also noted in the inaugural Insights Research Topic, there is a movement toward and appreciation for the need for studies and interventions that target middle age, when brain and cognitive alterations begin to change and arresting or reversing decline is more likely to be achieved.

## Author contributions

KN: Conceptualization, Writing – original draft, Writing – review & editing. AV: Writing – review & editing. SM: Writing – review & editing.
